# Five new *Caenorhabditis* species from Indonesia provide exceptions to Haldane's rule and partial fertility of interspecific hybrids

**DOI:** 10.1093/g3journal/jkaf134

**Published:** 2025-06-21

**Authors:** Mia Prastika Devi, Elkana Haryoso, Emha Ilhami Rais, Anggik Karuniawan, Minhajul Qowim Yahya, Aurélien Richaud, John Wang, Matthew V Rockman, Hagus Tarno, Marie-Anne Félix

**Affiliations:** Department of Pests and Disease, Faculty of Agriculture, University of Brawijaya, JL Veteran Malang, Malang, East Java 65145, Indonesia; Department of Pests and Disease, Faculty of Agriculture, University of Brawijaya, JL Veteran Malang, Malang, East Java 65145, Indonesia; Department of Pests and Disease, Faculty of Agriculture, University of Brawijaya, JL Veteran Malang, Malang, East Java 65145, Indonesia; Department of Pests and Disease, Faculty of Agriculture, University of Brawijaya, JL Veteran Malang, Malang, East Java 65145, Indonesia; Department of Pests and Disease, Faculty of Agriculture, University of Brawijaya, JL Veteran Malang, Malang, East Java 65145, Indonesia; Institut de Biologie de l’École Normale Supérieure, Centre National de la Recherche Scientifique, Institut National de la Santé et de la Recherche Médicale, École Normale Supérieure, Paris Sciences et Lettres, 46 rue d'Ulm Paris 75005, France; Biodiversity Research Center, Academia Sinica, Taipei 115, Taiwan; Department of Biology, Center for Genomics and Systems Biology, New York University, New York, NY 10003, USA; Department of Pests and Disease, Faculty of Agriculture, University of Brawijaya, JL Veteran Malang, Malang, East Java 65145, Indonesia; Institut de Biologie de l’École Normale Supérieure, Centre National de la Recherche Scientifique, Institut National de la Santé et de la Recherche Médicale, École Normale Supérieure, Paris Sciences et Lettres, 46 rue d'Ulm Paris 75005, France

**Keywords:** *Caenorhabditis*, nematode, biodiversity, Indonesia, Haldane's rule, speciation, Animalia, WormBase

## Abstract

Given the interest in the biogeography and diversity of the *Caenorhabditis* genus, we established a collection of these nematodes from field surveys on 4 Indonesian islands. We isolated over 60 *Caenorhabditis* strains belonging to 10 species. Five species were previously known from other locations: *Caenorhabditis briggsae*, which was predominant, *Caenorhabditis tropicalis*, *Caenorhabditis nigoni*, *Caenorhabditis brenneri*, and *Caenorhabditis elegans*. The 5 other species are new discoveries for science, and we describe them here as *Caenorhabditis indonesiana*, *Caenorhabditis malinoi*, *Caenorhabditis ceno*, *Caenorhabditis brawijaya*, and *Caenorhabditis ubi*. RNA sequence analysis of 1,861 orthologous genes placed all species from Indonesia in the *Elegans* group of *Caenorhabditis* species. Four of the new species belong to a *Sinica* subclade of species so far only found in an East Asia-Indo-Pacific world region. The fifth new species, *C. indonesiana*, appears as the sister of the *C. tropicalis*–*Caenorhabditis wallacei* pair, both also found in Indonesia. The present findings are thus consistent with diversification in the *Elegans* group having occurred in this world region. Crosses between closely related species showed counterexamples to Haldane's “rule”: for several pairs of species, in one cross direction, we only found hybrid males. In addition, we found a pair of species that could partially interbreed: *C. ubi* (East Java) with *C.* sp. 41 (Solomon islands), with the hybrid males in one cross direction being fertile. Such closely related species pairs are good models for genetic studies of incompatibilities arising during speciation.

## Teaser

This work addresses the biodiversity, phylogenetic relationships, and genetic incompatibilities of *Caenorhabditis* nematodes, which are laboratory model organisms. Through field studies, the authors isolated 60 *Caenorhabditis* strains in Indonesia, representing ten species, including five newly described. From RNA sequencing and phylogenetic reconstruction, all ten species belong to the *Elegans* group of *Caenorhabditis*. In crosses between closely related species, the hybrid progeny can be all females, abiding by Haldane’s rule, but in other cases all males. In one species pair, partially fertile hybrids are produced in one cross direction. These closely related species are good models for studying genetic incompatibilities.

## Introduction

*Caenorhabditis elegans* is a free-living nematode species that is widely used as a biological model organism. Building a solid evolutionary framework of genetic and phenotypic variation within and around this model species is thus important for providing a context for its biology and as a resource for evolutionary biology and ecology. The last 20 years have seen a blossom of discovery of new *Caenorhabditis* species (Kiontke *et al*. 2011; Félix *et al*. 2014; Huang *et al*. 2014; Ferrari *et al*. 2017; Slos *et al*. 2017; Kanzaki *et al*. 2018; Crombie *et al*. 2019; Stevens *et al*. 2019; Sloat *et al*. 2022). Many species including *C. elegans* can be found in rotting vegetal material rich in bacteria, such as decomposing fruits, flowers, and stems (Kiontke *et al*. 2011; Schulenburg and Félix 2017). These species, especially their larval diapause stage called the dauer larva, can be carried between food patches by larger invertebrates such as mollusks and arthropods (Félix and Braendle 2010).

The present phylogeny and biogeography of the *Caenorhabditis* genus indicate that the *Elegans* group of species may have radiated in the Asia-Pacific region (Frézal and Félix 2015; Stevens *et al*. 2019). The most divergent populations of *C. elegans* were found in the Hawaiian Islands and the rim of the Pacific Ocean (Crombie *et al*. 2019; Lee *et al*. 2021; Crombie *et al*. 2024). The present sister species of *C. elegans*, named *Caenorhabditis inopinata*, was found in South Japan on tropical figs and their associated wasps (Kanzaki *et al*. 2018; Woodruff and Phillips 2018). In Indonesia, another fig-associated species provisionally called *Caenorhabditis* sp. 35 was found in West Sumatra (Jauharlina *et al*. 2022), and an *Elegans* group species, *C. wallacei,* was described from Bali (Kiontke *et al*. 2011; Félix *et al*. 2014). The sampling of *Caenorhabditis* strains from Indonesia was thus likely to yield new species, as well as insights about their habitat and diversity.

Most *Caenorhabditis* species reproduce through males and females. Only 3 species, *C. elegans*, *Caenorhabditis briggsae*, and *Caenorhabditis tropicalis*, independently evolved a mode of reproduction via selfing hermaphrodites and facultative males (Kiontke *et al*. 2011). Finding a closer sister species to *C. elegans* would be valuable. In addition, pairs of species that are partially cross-fertile, such as *C. briggsae*–*Caenorhabditis nigoni* (Woodruff *et al*. 2010; Bi *et al*. 2015; Bundus *et al*. 2015) or *Caenorhabditis latens*–*Caenorhabditis remanei* (Dey *et al*. 2012), provide good cases for studies of genetic incompatibilities arising during speciation (Cutter 2018).

Given the interest in the biogeography of the *Caenorhabditis* genus, the quest for closely related species to study speciation mechanisms and the paucity of previous sampling, we aimed to collect and culture *Caenorhabditis* from Indonesia. Diverse elevations and landscape types (e.g. forests, agricultural land) and various sample types (rotting vegetal and fungal matter, such as flowers, fruits, stems, leaves, wood, fungi) were collected. We found 11 different species and could maintain 10 in culture, 5 of which are newly discovered. All are in the *Elegans* group of *Caenorhabditis* species. Interestingly, some crosses between closely related species only yielded F1 hybrid males, providing counterexamples to Haldane's rule. In addition, we found a pair of species (*Caenorhabditis ubi* n. sp. and *C.* sp. 41 from the Solomon Islands) that could partially interbreed.

## Materials and methods

### Sampling and isolation

Using a field survey method, locations were selected based on suitability with the habitat of nematodes of the genus *Caenorhabditis* in decomposing vegetation. Sampling of diverse substrates, mostly decomposing plant matter (Supplementary Table 1), was conducted across several regions in Indonesia, at various points on Java, Sulawesi, Lombok, and Bali islands, in forests, agroforestry, agricultural landscapes, and parks. Each observation location was marked using a Global Positioning System (GPS) to record the altitude and coordinates of the observation points. On Java Island, samples were collected from Coban Talun in Wonorejo Hamlet, Tulungrejo Village, Bumiaji District, Batu City; University of Brawijaya Forest in Tawang Agro Village, Karangploso Sub-district, Malang Regency; and Mount Bromo in Ngadas Village, Sukapura Sub-district, Probolinggo Regency. On Sulawesi Island, sampling was carried out in 2 villages in Gowa Regency, Central Sulawesi Province, Malino and Lanna Villages. From Lombok Island, West Nusa Tenggara Province, samples were collected from Lingsar Village in West Lombok Regency and Setiling Village in Central Lombok Regency. From Bali Island, samples were collected in Sayan, Ubud, Megwi, Marga, Ababi, and Besakih. The extraction and identification processes were conducted at the Laboratory of Plant Pathology I and Laboratory of Plant Pest II, Department of Plant Pests and Diseases, Faculty of Agriculture, Universitas Brawijaya, Malang, East Java, Indonesia, and at the Institut de Biologie de l'Ecole Normale Supérieure (IBENS), Paris, France.

For nematode extraction and culture in Malang, we used Petri dishes containing nematode growth medium (NGM) with *Escherichia coli*  OP50 placed at the center. The preparation began by making the NGM. A total of 14.5 g of NGM were added to a 1-L Schott bottle, followed by 500 mL of distilled water. The Schott bottle was then placed in a clear polypropylene plastic bag and autoclaved at 121°C and 20 psi pressure for an hour. After autoclaving, the bottle was allowed to cool for 10 min before the medium was aseptically poured into glass or plastic Petri dishes of 90 or 55 mm diameter for extraction and culture, respectively. Once the medium solidified, 100 μL of *E. coli*  OP50 culture was added to the center of each dish using a micropipette. The pouring of NGM and addition of *E. coli*  OP50 culture were conducted in a laminar air flow cabinet to prevent contamination. The *E. coli* on the NGM was incubated for 24 h at room temperature.

Supplementary Table 1 provides the time of field collection and of plating the samples in the laboratory. The plating of samples was performed in a time interval varying from 1 day to 2 weeks for samples analyzed in Paris. This study employed the Agar Culture Plate extraction method (Barrière and Félix 2014). Approximately 10 g samples of substrate were collected, with the plant samples being cut into smaller pieces using scissors. These samples were then evenly spread in a circular pattern along the edge of the NGM plate. The plates were left at room temperature to allow nematodes to migrate from the substrate toward the *E. coli*  OP50 on the NGM. Each plate was monitored several times (from a few hours to a few days) and nematodes that migrated to *E. coli* were transferred aseptically using a nematode platinum wire pick to a fresh *E. coli*  OP50-seeded NGM plate. The nematodes initially transferred to establish an isofemale line were single hermaphrodites, single mated females, or a pair of nematodes (male and female), ensuring that the population contained a single species.

### Culture and freezing

Isofemale strains were maintained and frozen using standard methods. The nematodes cultured on NGM plates were transferred to fresh plates either using the pick or the “Chunking” method (Stiernagle 2006). This latter method involved cutting a section of agar from the old NGM plate using a sterilized syringe or spatula and transferring the agar piece to a new NGM plate.

Nematodes were frozen in Paris with standard *C. elegans* protocols (Stiernagle 2006). All strains thrive at 25°C, except *C. elegans* HPT48, and *C. brawijaya* n. sp. HPT49 and HPT50, which grow better at 20°C.

### Mode of reproduction, crosses, and assignment of strains to biological species

The mode of reproduction of each strain was assessed by isolation of L4 larvae with a female body. In these circumstances, selfing hermaphrodites produce progeny, and females fail to produce progeny.

Crosses between male–female strains were established by placing together 5 L4 females of one strain and 5 L4 to adult males of another strain. The plate was checked for the presence of cross-progeny (F1) and later cross-fertility of the F1 animals over several days. In some cases, backcrosses of the F1 animals to the parental strains were performed.

Species with selfing XX hermaphrodites produce rare X0 males that can be crossed like *C. elegans*. For crosses with these species, selfing hermaphrodites were crossed to males, also using 5 animals of each sex. A cross was considered positive when the progeny included a high proportion of males (>20%).

All strains and their geographic position can be found and visualized on a world map on the following website: https://justbio.com/tools/worldwideworms/search.php.

### Internal transcribed sequence 2 sequencing

Sequencing of the ribosomal DNA internal transcribed spacer ITS2 (internal transcribed sequence 2) was performed as in Kiontke *et al*. (2011), using primers 5.8S-1 and KK28S-22 for amplification and KK28S-22 for Sanger sequencing. For the ITS2 sequences that were highly polymorphic (HPT35 and HPT43), the fragments were cloned into the pGEM-T easy vector (Promega) and sequenced with a T7 primer. The ITS2 sequences are deposited at GenBank with accession numbers PV569245–PV569249.

### RNA sequencing

Before RNA preparation, the reference strains HPT10, HPT35, and HPT5 were inbred by crossing a single L4 female and a male (brother–sister mating) for 5 generations. HPT43 was further inbred for 25 generations yielding inbred strain JU4643. HPT50 was used without inbreeding. RNA was prepared from a mixed-stage population using Trizol and a freeze–thaw cycle as in Stevens *et al*. (2019). Sequencing libraries were prepared from polyA RNA and sequenced on an Illumina NovaSeq with 150 bp paired-end reads.

### Phylogeny

We estimated the phylogeny of the *Elegans* group from the amino acid sequences of single-copy proteins, following the procedure in Sloat *et al*. (2022). First, RNAseq read-pairs were trimmed using TrimGalore 0.6.6 (https://github.com/FelixKrueger/TrimGalore) in paired mode with *q* = 25, and transcriptomes assembled using Trinity 2.15.1 (Grabherr *et al*. 2011) with default settings. The longest isoform for each transcript was extracted with TransDecoder 5.5.0 (https://github.com/TransDecoder/TransDecoder) and the resulting transcriptomes processed through BUSCO 5.3.0 (Seppey *et al*. 2019) to identify genes representing 3131 core gene models in the nematoda_odb10 database. We next collected analogous data from an additional 21 *Elegans* group species and used busco2fasta.py (https://github.com/lstevens17/busco2fasta) to collect sets of BUSCO genes present as single-copy genes in the transcriptomes at least 80% of the species. The predicted amino acid sequences of each gene were then aligned using MAFFT 7.475 (Katoh and Standley 2013) with automatic parameter selection, and the alignments trimmed to alignable positions using trimAl 1.4.1 (Capella-Gutierrez *et al*. 2009) with settings gt 0.8, st 0.001, resoverlap 0.75, and seqoverlap 80. We estimated the gene tree for each of the 1,861 protein alignments using iqtree 1.6.12 (Nguyen *et al*. 2015) under the LG + I + G substitution model. We estimated the species tree from the collection of gene trees using Astral 5.7.8 (Zhang *et al*. 2018), and we estimated branch lengths for the species tree using iqtree -te with the concatenation of all of the protein sequence alignments (755,373 amino acid positions), which we generated with catfasta2phyml (https://github.com/nylander/catfasta2phyml). Plots were generated with the ape package (Paradis and Schliep 2019). RNAseq data and assembled transcriptomes are available under NCBI BioProject PRJNA1256413. The computational pipeline is provided in Supplementary File 1. The species tree is provided in Newick format in Supplementary File 2.

### Morphology and Nomarski micrographs

Pictures of whole animals on the agar plates were taken using a Nikon Multizoom AZ100 equipped with a Hamamatsu Orca-flash 4.0 camera. Morphology was observed under Nomarski microsopic illumination with a 100× objective on an AxioImager 2 (Zeiss), after mounting the animals on a Noble agar pad (Shaham 2006). Pictures were taken using a Photometrics CoolSNAP ES CCD camera. Pictures showing extruded spicules were obtained after exposing the animals for 2–4 s in a microwave before adding the coverslip.

## Results

### Field sampling of *Caenorhabditis*

**Fig. 1.**
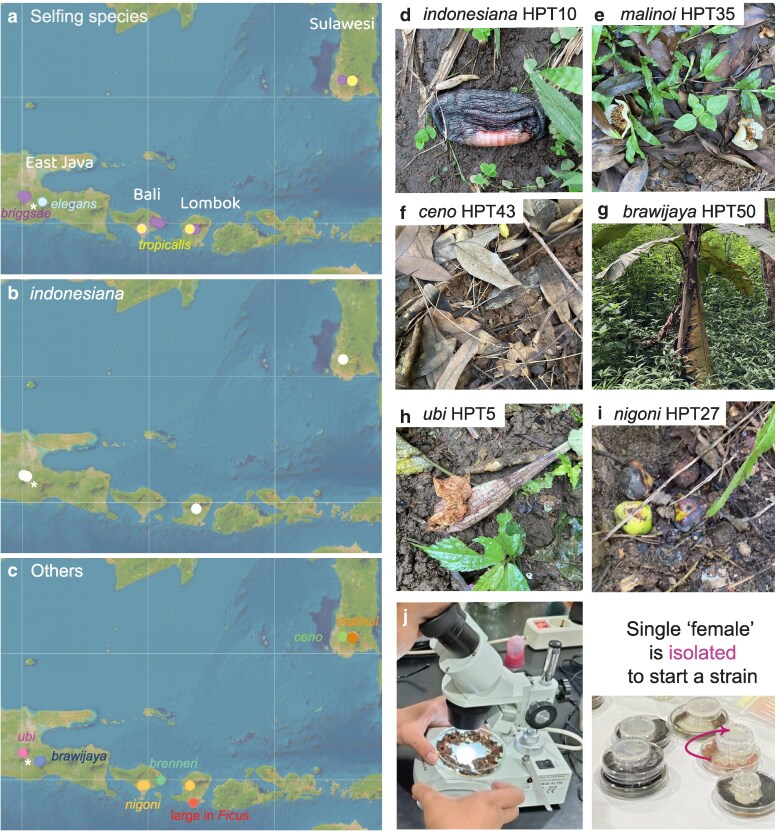
Geographic distribution of *Caenorhabditis* collected in Indonesia. a) Geographic distribution of the 3 selfing *Caenorhabditis* species. *Caenorhabditis briggsae* is found at most sampling sites. b) Geographic distribution of *C. indonesiana* (sp. 77) on several islands. c) Geographic distribution of other species. The species are color-coded. The asterisk designates the city of Malang. Map credit: ESRI. d–i) Pictures of representative samples. Shown are the 5 samples that yielded the reference strain of each newly discovered species and a rotting fruit that yielded *C. nigoni*. d) HPT10: decomposing banana flower. e) HPT35: *S. pseudocamellia* flowers in various stages of decomposition. f) HPT43: forest leaf litter. g) HPT50: decomposing *Musa* pseudostem. h) HPT5: decomposing *Brugmansia* flower. j) Procedure for *Caenorhabditis* isolation. The samples are brought back to the laboratory and placed on agar plates (diameter 90 mm) seeded with *E. coli* bacteria. They are examined under a dissecting microscope. Single females or hermaphrodites are picked to a smaller plate (diameter 55 mm) to start an isofemale line.

We sampled on 4 islands, Java, Bali, Lombok, and Sulawesi (Fig. 1), at different elevations between sea level and 2,400 m. We collected samples of decomposing vegetal matter and a few invertebrates, such as mollusks, annelids, and arthropods (Supplementary Table 1).

Out of 204 samples collected in April–May 2024 (Table 1 and Supplementary Table 1), 58 were positive for *Caenorhabditis* and some samples yielded 2 or 3 *Caenorhabditis* species. Positive samples included mostly decomposing plant material (fruits, flowers, stems, leaves), as well as fresh *Ficus septica* figs and a snail. From these positive samples, we established 61 isofemale strains that could be maintained in long-term culture and frozen. In addition, a sample of fresh *F. septica* figs yielded a large *Caenorhabditis* species that could only be maintained for few generations; these animals could be *C. inopinata* (Kanzaki *et al*. 2018; Woodruff and Phillips 2018) or the species isolated in Jauharlina *et al*. (2022). One additional strain (HPT1) was isolated a few weeks earlier from an apple orchard. Out of the 62 strains in long-term culture, 37 reproduced by selfing of hermaphrodites and 25 through males and females.

**Table 1.** Summary of samples, strains, species, locations.

Location	SA	PO	ST	*Cel*	*Cbr*	*Ctr*	*Cbn*	*Cni*	77 *Cid*	78 *Cml*	79 *Cce*	80 *Cbw*	81 *Cub*	*Cin* or sp. 35?
Batu forest, East Java	25	11	11	—	8	—	—	—	2	—	—	—	1	—
UB Forest, East Java	41	8	10	—	6	—	—	—	4	—	—	—	—	—
Bromo area, East Java	18	3	3	1	—	—	—	—	—	—	—	2	—	—
Lombok	46	10	10	—	3	2	—	2	2	—	—	—	—	1
South Sulawesi	40	13	15	—	4	2	—	—	1	5	3	—	—	—
Bali	34	13	13	—	9	1	1	2	—	—	—	—	—	—
Total	204	58	62	1	30	5	1	4	9	5	3	2	1	(1)

This table provides the number of samples (SA), positive samples (PO), strains (ST), and strains of each species. *Cel*, *C. elegans*; *Cbr*, *C. briggsae*; *Ctr*, *C. tropicalis*; *Cbn*, *C. brenneri*; *Cni*, *C. nigoni*; *Cin*, *C. inopinata*. See Table 2 for the names of the 5 newly discovered species (columns labeled 77–81) and their abbreviations. The strain in the last column could not be maintained after a few generations. An additional *C. briggsae* strain (HPT1) was found near Batu. Details can be found in Supplementary Table 1. UB, Universitas Brawijaya.

### Selfing *Caenorhabditis* species

From the results of our crosses, all selfing strains belonged to one of the 3 known selfing *Caenorhabditis* species. Specifically, the majority (31/37) were *C. briggsae* and this was true on all sampled islands (Fig. 1 and Table 1). Five strains belonged to *C. tropicalis*, found in Lombok, South Sulawesi, and Bali, but this species was absent from the East Java sites, possibly because of their higher elevation (Supplementary Table 1). Finally, a single *C. elegans* isolate was found, in a sample collected at an elevation of 2,150 m next to the Bromo volcano in East Java, in a volcanic ash landscape with a few low bushes (Supplementary Fig. 1).

### Male–female *Caenorhabditis* species

We first crossed the male–female *Caenorhabditis* strains from Indonesia to each other to group them into cross-compatible biological species. This defined 7 biological species (Supplementary Table 2). Crosses of representative strains of each group to *C. wallacei*, which had been previously isolated from Bali, were all negative. We then sequenced a PCR product corresponding to the ITS2 of ribosomal DNA. Based on high ITS2 sequence similarity, we set up crosses for 2 of them with *C. nigoni* or *Caenorhabditis brenneri*, respectively, and both crosses were successful (Supplementary Table 2). Both *C. nigoni* and *C. brenneri* are cosmopolitan in tropical areas of the world. In the present collection, we found 1 *C. brenneri* in Bali and 4 *C. nigoni* strains, 2 in Lombok, and 2 in Bali (Table 1). *Caenorhabditis nigoni* is partially cross-fertile with *C. briggsae* (Woodruff *et al*. 2010; Bi *et al*. 2015; Bundus *et al*. 2015) and we note that both could be found in the same sampling locations (Supplementary Table 1).

**Fig. 2.**
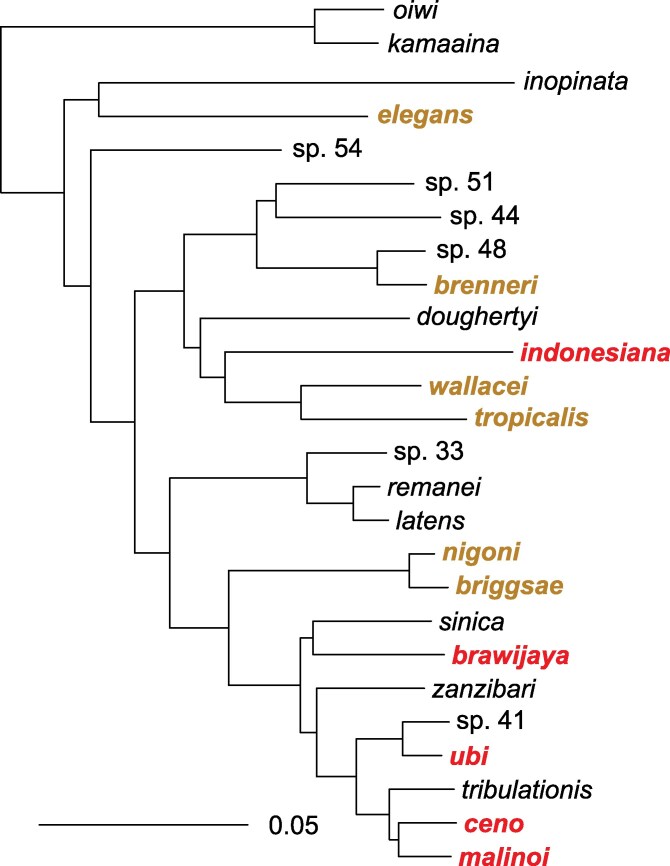
Phylogenetic relationships of the *Elegans* group of *Caenorhabditis* species. New species are in red; other species found in this study or previously in Indonesia are in brown. The topology was estimated under the multispecies coalescent from 1,861 maximum-likelihood gene trees, and is shown rooted with the outgroup species *Caenorhabditis oiwi* and *Caenorhabditis kamaaina*. The scale bar represents 0.05 amino acid replacements per site. Quadripartition support values are 1 for all branches. The tree in Newick format can be found in Supplementary File 2.

The 5 other species appeared novel from the ITS2 sequence. All of them were closest to species in the *Elegans* group in the clade including *C. briggsae* and *C. brenneri* but not *C. elegans*. We sequenced RNA from a representative strain of each of these 5 species and assembled transcriptomes from the reads. Using protein sequences of 1,861 single-copy genes for 26 species within the *Elegans* group, we estimated the phylogenetic position of the Indonesian species (Kiontke *et al*. 2011; Stevens *et al*. 2019; Supplementary Table 3 and Fig. 2).

**Fig. 3.**
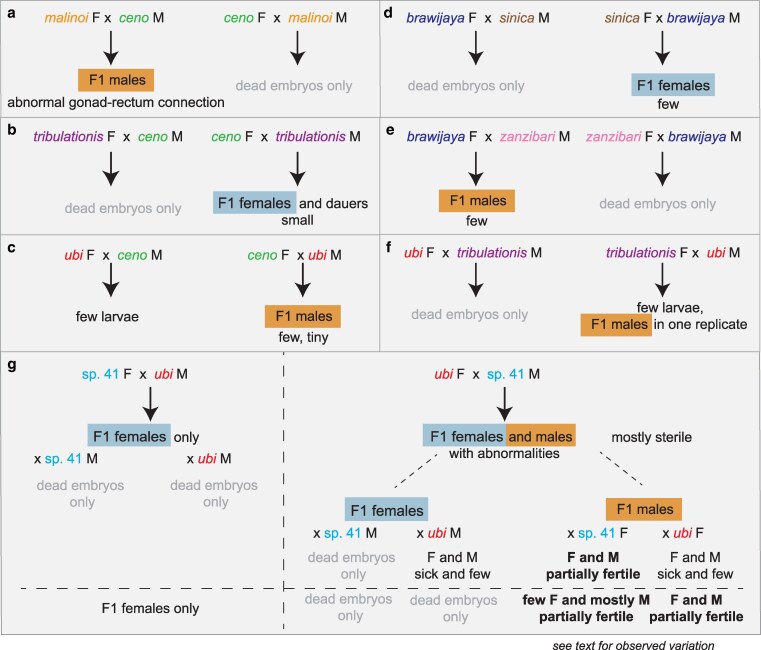
Noteworthy crosses between *Caenorhabditis* species. a–f) Crosses that could yield F1 adults of 1 sex, but no further generations, while g) shows the crosses involving the 2 species that can yield some progeny in further generations. Some representative pictures are shown in Figs. 4 and 5, respectively. F, female; M, male. Detailed and additional cross results are found in Supplementary Table 2. Crosses may have variable results depending on the replicate experiment. In crosses after a new thaw of the *C. ubi* and *C.* sp. 41 strains (below the dash line in g), the fertile F1 males gave rise to mostly males when crossed to *C.* sp. 41 females and to partially fertile males and females when crossed to *C. ubi f*emales, which could allow a population to propagate.

One of them, represented by strain HPT10, was present on several islands, matched *C. wallacei* most closely (Supplementary Table 4) but could not interbreed with it and thus defined a newly discovered species. The 4 others were each restricted to a single island in our collection and were in a subclade including *Caenorhabditis sinica*, *Caenorhabditis zanzibari*, *Caenorhabditis tribulationis*, and *C.* sp. 41 (represented by strain BRC20276). We crossed representative strains of each group to described species that were closest to them. Some crosses yielded larval or adult progeny but did not produce a normally fertile brood (Fig. 3 and Supplementary Table 2). We thus raise here 5 new species.

### Species declarations

The electronic edition of this article conforms to the requirements of the amended International Code of Zoological Nomenclature, and hence, the new names contained herein are available under that Code from the electronic edition of this article. This published work and the nomenclatural acts it contains have been registered in ZooBank, the online registration system for the ICZN. The ZooBank LSIDs (Life Science Identifiers) can be resolved and the associated information viewed through any standard web browser by appending the LSID to the prefix “http://zoobank.org/.” The LSID for this publication is: urn:lsid:zoobank.org:pub:68091B60-E71A-4196-92E7-6033E400AD8C. The electronic edition of this work was published in a journal with an ISSN.

Most *Caenorhabditis* species in the *Elegans* group are highly similar morphologically; therefore, we define them solely based on the biological species concept. Crosses were prioritized using genetic proximity (Félix *et al*. 2014). We provide pictures of their male tail in Supplementary Fig. 2 to conform with the ICZN guidelines.


$$\begin{array}{rl} & Caenorhabditis\;indonesiana\;\mathrm{Tarno}\;\mathrm{and}\;Fé\mathrm{lix}\;\mathrm{sp}.\;n.\\ & \mathrm{Zoobank}\;\mathrm{identifier}\\ & \;\;\;\mathrm{urn}:\mathrm{lsid}:\mathrm{zoobank}.\mathrm{org}:\mathrm{act}:6D761875\text{-}0\mathrm{EC}7\text{-}45D6\\ & \;\;\;\text{-}\mathrm{AD}06\text{-}749\mathrm{DB}4A0B124\\ & =Caenorhabditis\;\mathrm{sp}.\;77\;(\mathrm{temporary}\;\mathrm{number}) \end{array}$$

The type isolate by present designation is HPT10. This strain is derived from a single female. The strain thus includes a single species. The holotype is deposited at the *Caenorhabditis* Genetics Center under the strain name HPT10 and paratypes are deposited as frozen strains at the Museum Koenig Bonn under ID#ZFMK-TIS-99298 to #ZFMK-TIS-99303. The species reproduces through males and females. The species is delineated and diagnosed by the fertile cross with the type isolate HPT10 in both cross directions, yielding highly fertile hybrid females and males that are interfertile and cross-fertile with their parent strains. This species differs by ITS2 DNA sequence from all species listed in Tables 1 and 2 of Félix *et al*. (2014), those described in Huang *et al*. (2014), Ferrari *et al*. (2017), Slos *et al*. (2017), Kanzaki *et al*. (2018), Crombie *et al*. (2019), Stevens *et al*. (2019) , Dayi *et al*. (2021), Sloat *et al*. (2022), and other species in Table 2 of the present article. Note that these ribosomal DNA sequences may vary among repeated copies and within the species. From RNA sequence data, the closest species is *C. wallacei*, with which it does not form any adult progeny (Supplementary Table 2). The type isolate was collected from a rotting banana flower collected in a forest near Batu, East Java, Indonesia (GPS −7.803387, 112.516604) on 2024 April 28. Other isolates were found in decomposing vegetal matter in East Java, Lombok, and South Sulawesi, Indonesia (Supplementary Table 1). The fan of the male tail is wide (Supplementary Fig. 2). Dorsal rays are found in anteroposterior positions 5 and 7. The males mate in a parallel position. The species is named after its place of isolation.


$$\begin{array}{rl} & Caenorhabditis\;malinoi\;\mathrm{Tarno}\;\mathrm{and}\;Fé\mathrm{lix}\;\mathrm{sp}.\;n.\\ & \mathrm{Zoobank}\;\mathrm{identifier}\\ & \;\;\;\;\mathrm{urn}:\mathrm{lsid}:\mathrm{zoobank}.\mathrm{org}:\mathrm{pub}:68091B60\text{-}E71A\text{-}4196\text{-}92E7\\ & \;\;\;\;\text{-}6033E400\mathrm{AD}8C\\ & =Caenorhabditis\;\mathrm{sp}.\;78\;(\mathrm{temporary}\;\mathrm{number}) \end{array}$$

**Table 2.** Newly described *Caenorhabditis* species.

Species	Temporary number	Type strain	Mode of reproduction	3-Letter abbreviation	RNAseq accession number	Transcriptome assembly accession
*indonesiana*	77	HPT10	Male–female	Cid	SAMN48179281	GLGD00000000
*malinoi*	78	HPT35	Male–female	Cml	SAMN48179282	GLGE00000000
*ceno*	79	HPT43	Male–female	Cce	SAMN48179283	GLGF00000000
*brawijaya*	80	HPT50	Male–female	Cbw	SAMN48179284	GLGG00000000
*ubi*	81	HPT5	Male–female	Cub	SAMN48179285	GLGC00000000

*indonesiana*, Indonesian. *malinoi*, of Malino. *ceno*: from the mud (Latin). *brawijaya*, Java prince and name of the university. *ubi*, for Universitas Brawijaya (UB). Three-letter abbreviations avoid repeating that used for other *Caenorhabditis* species. Alternatively, 4 letters can be used.

The type isolate by present designation is HPT35. This strain is derived from a single female. The strain thus includes a single species. The holotype is deposited at the *Caenorhabditis* Genetics Center under the strain name HPT35 and paratypes are deposited as frozen strains at the Museum Koenig Bonn under ID#ZFMK-TIS-99304 to #ZFMK-TIS-99309. The species reproduces through males and females. The species is delineated and diagnosed by the fertile cross with the type isolate HPT35 in both cross directions, yielding highly fertile hybrid females and males that are interfertile and cross-fertile with their parent strains. This species differs by ITS2 DNA sequence from all species listed in Tables 1 and 2 of Félix *et al*. (2014), those described in Huang *et al*. (2014), Ferrari *et al*. (2017), Slos *et al*. (2017), Kanzaki *et al*. (2018), Crombie *et al*. (2019), Stevens *et al*. (2019), Dayi *et al*. (2021), Sloat *et al*. (2022), and other species in Table 2 of the present article. Note that these ribosomal DNA sequences may vary among repeated copies and within the species. From RNA sequence data, the closest species are those in the clade formed by *C. zanzibari*, *C. tribulationis*, *C. sinica*, *C.* sp. 41, and the species described in the present article as *Caenorhabditis ceno*, *C. brawijaya*, and *C. ubi*. The closest species is *C. ceno*. It does not form fertile hybrids with any of these species. *Caenorhabditis malinoi* females crossed to *C. ceno* males produce sterile males (Supplementary Table 2). The type isolate was collected from rotting flowers of *Stewartia pseudocamellia* collected in a forest park in Malino, South Sulawesi, Indonesia (GPS −5.242944, 119.868592) on 2024 May 6. Other isolates were found in rotting fruits in other locations around Malino (Supplementary Table 1). The fan of the male tail is wide (Supplementary Fig. 2). Dorsal rays are found in anteroposterior positions 5 and 7. The anterior side of the hook shows a distinctive 3-lobed shape, which is a shared character with *C. sinica* (Huang *et al*. 2014), *C*. *tribulationis*, and *C. zanzibari* (Stevens *et al*. 2019). The males mate in a parallel position. The species is named after its place of isolation.


$$\begin{array}{rl} & Caenorhabditis\;ceno\;\mathrm{Tarno}\;\mathrm{and}\;Fé\mathrm{lix}\;\mathrm{sp}.\;n.\\ & \mathrm{Zoobank}\;\mathrm{identifier}\\ & \;\;\mathrm{urn}:\mathrm{lsid}:\mathrm{zoobank}.\mathrm{org}:\mathrm{act}:C3A6D80C\text{-}9B1F\text{-}45A1\text{-}A2A4\\ & \;\;\text{-}38\mathrm{FC}8A38F132\\ & =Caenorhabditis\;\mathrm{sp}.\;79\;(\mathrm{temporary}\;\mathrm{number}) \end{array}$$

The type isolate by present designation is HPT43. This strain is derived from a single female. The strain thus includes a single species. The holotype is deposited at the *Caenorhabditis* Genetics Center under the strain name HPT43 and paratypes are deposited as frozen strains at the Museum Koenig Bonn under ID#ZFMK-TIS-99310 to #ZFMK-TIS-99314. The species reproduces through males and females. The species is delineated and diagnosed by the fertile cross with the type isolate HPT43 in both cross directions, yielding highly fertile hybrid females and males that are interfertile and cross-fertile with their parent strains. This species differs by ITS2 DNA sequence from all species listed in Tables 1 and 2 of Félix *et al*. (2014), those described in Huang *et al*. (2014), Ferrari *et al*. (2017), Slos *et al*. (2017), Kanzaki *et al*. (2018), Crombie *et al*. (2019), Stevens *et al*. (2019), Dayi *et al*. (2021), Sloat *et al*. (2022), and other species in Table 2 of the present article. Note that these ribosomal DNA sequences may vary among repeated copies and within the species. From RNA sequence data, the closest species are those in the clade formed by *C. zanzibari*, *C. tribulationis*, *C. sinica*, *C.* sp. 41, and the species described in the present article as *C. malinoi*, *C. brawijaya*, and *C. ubi*. The closest species is *C. malinoi*. It does not form fertile hybrids with any of these species. *Caenorhabditis ceno* males crossed to *C. malinoi* females produce sterile males. *Caenorhabditis ceno* females crossed to *C. ubi* males produce small hybrid males and to *C. tribulationis* males produce small hybrid females (Supplementary Table 2). The type isolate was collected from leaf litter collected in a forest in South Sulawesi, Indonesia (GPS −5.238297, 119.642281) on 2024 May 6. Two other isolates were found in the same forest (Supplementary Table 1). The fan of the male tail is wide (Supplementary Fig. 2). Dorsal rays are found in anteroposterior positions 5 and 7. The anterior side of the hook shows a distinctive 3-lobed shape that is shared with *C. sinica* (Huang *et al*. 2014), *C*. *tribulationis*, and *C. zanzibari* (Stevens *et al*. 2019). The males mate in a parallel position. The species is named after the mud in the forest where it was collected (from the Latin cenum).


$$\begin{array}{rl} & Caenorhabditis\;brawijaya\;\mathrm{Tarno}\;\mathrm{and}\;Fé\mathrm{lix}\;\mathrm{sp}.\;n.\\ & \mathrm{Zoobank}\;\mathrm{identifier}\\ & \;\;\;\mathrm{urn}:\mathrm{lsid}:\mathrm{zoobank}.\mathrm{org}:\mathrm{act}:3058D518\text{-}A8\mathrm{DA}\text{-}44\mathrm{DA}\text{-}90\mathrm{FC}\\ & \;\;\;\text{-}\mathrm{DD}826A5D19A7\\ & =Caenorhabditis\;\mathrm{sp}.\;80\;(\mathrm{temporary}\;\mathrm{number}) \end{array}$$

The type isolate by present designation is HPT50. This strain is derived from a single female. The strain thus includes a single species. The holotype is deposited at the *Caenorhabditis* Genetics Center under the strain name HPT50 and paratypes are deposited as frozen strains at the Museum Koenig Bonn under ID#ZFMK-TIS-99315 to #ZFMK-TIS-99319. The species reproduces through males and females. The species is delineated and diagnosed by the fertile cross with the type isolate HPT50 in both cross directions, yielding highly fertile hybrid females and males that are interfertile and cross-fertile with their parent strains. This species differs by ITS2 DNA sequence from all species listed in Tables 1 and 2 of Félix *et al*. (2014), those described in Huang *et al*. (2014), Ferrari *et al*. (2017), Slos *et al*. (2017), Kanzaki *et al*. (2018), Crombie *et al*. (2019), Stevens *et al*. (2019), Dayi *et al*. (2021), Sloat *et al*. (2022), and other species in Table 2 of the present article. Note that these ribosomal DNA sequences may vary among repeated copies and within the species. From RNA sequence data, the closest species are the *C. zanzibari*, *C. tribulationis*, *C. sinica*, *C.* sp. 41, and the species described in the present article as *C. malinoi*, *C. ceno*, and *C. ubi*. The closest and present sister species is *C. sinica. Caenorhabditis brawijaya* does not form fertile hybrids with any of these species but males crossed to *C. sinica* yielded some adult female progeny and females crossed to *C. zanzibari* males produce some sterile males (Supplementary Table 2). The type isolate was collected from a rotting *Musa* pseudostem collected in a forest near Ngadas, Malang Regency, East Java, Indonesia (GPS −7.99746, 112.87387) on 2024 May 11. Another isolate was found in decomposing leaves a few kilometers away (Supplementary Table 1). The fan of the male tail is wide (Supplementary Fig. 2). Dorsal rays are found in anteroposterior positions 5 and 7. The anterior side of the hook shows a distinctive 3-lobed shape that is shared with *C. sinica* (Huang *et al*. 2014), *C*. *tribulationis*, and *C. zanzibari* (Stevens *et al*. 2019). The males mate in a parallel position. The species is named after the Javanese prince after whom the Universitas Brawijaya is named.


$$\begin{array}{rl} & Caenorhabditis\;ubi\;\mathrm{Tarno}\;\mathrm{and}\;Fé\mathrm{lix}\;\mathrm{sp}.\;n.\\ & \mathrm{Zoobank}\;\mathrm{identifier}\\ & \;\;\mathrm{urn}:\mathrm{lsid}:\mathrm{zoobank}.\mathrm{org}:\mathrm{act}:2B4292\mathrm{DE}\text{-}2F01\text{-}4E87\text{-}8E11\\ & \;\;\text{-}E82811024443\\ & =Caenorhabditis\;\mathrm{sp}.\;81\;(\mathrm{temporary}\;\mathrm{number}) \end{array}$$

The type isolate by present designation is HPT5. This strain is derived from a single female. The strain thus includes a single species. The holotype is deposited at the *Caenorhabditis* Genetics Center under the strain name HPT5 and paratypes are deposited as frozen strains at the Museum Koenig Bonn under ID#ZFMK-TIS-99320 to #ZFMK-TIS-99324. The species reproduces through males and females. The species is delineated and diagnosed by the fertile cross with the type isolate HPT5 in both cross directions, yielding highly fertile hybrid females and males that are interfertile and cross-fertile with their parent strains. This species differs by ITS2 DNA sequence from all species listed in Tables 1 and 2 of Félix *et al*. (2014), those described in Huang *et al*. (2014), Ferrari *et al*. (2017), Slos *et al*. (2017), Kanzaki *et al*. (2018), Crombie *et al*. (2019), Stevens *et al*. (2019), Dayi *et al*. (2021), and Sloat *et al*. (2022), and other species in Table 2 of the present article. Note that these ribosomal DNA sequences may vary among repeated copies and within the species. From RNA sequence data, the closest species are *C.* sp. 41, *C. zanzibari*, *C. tribulationis*, *C. sinica*, and the species described in the present article as *C. malinoi*, *C. ceno*, and *C. brawijaya*. It forms some fertile hybrids in some cross-directions with *C.* sp. 41 as described in the present article (Supplementary Table 2 and Fig. 3g). The type isolate was collected from a rotting banana flower collected near Batu, East Java, Indonesia (GPS −7.803387, 112.516604) on 2024 April 28 (Supplementary Table 1). The fan of the male tail is wide (Supplementary Fig. 2). Dorsal rays are found in anteroposterior positions 5 and 7. The anterior side of the hook shows a distinctive 3-lobed shape that is shared with *C. sinica* (Huang *et al*. 2014), *C*. *tribulationis*, and *C. zanzibari* (Stevens *et al*. 2019). The males mate in a parallel position. The species is named after the Universitas Brawijaya (UB).

### Phylogenetic relationships including closely related pairs of species

From our phylogenetic reconstruction using RNA sequencing of 1,861 orthologous single-copy genes (Fig. 2), 4 of these newly described species belong to a *Sinica* subclade of species so far only found in an East Asia-Indo-Pacific world region. This clade includes 2 pairs of particularly closely related species with short branches on the tree and low distances (Fig. 2 and Supplementary Table 4): *C. ubi* with *C.* sp. 41 from the Solomon Islands, and *C. ceno* with *C. malinoi*, the latter 2 both from 30 km apart in South Sulawesi.

The fifth species, *Caenorhabditis indonesiana*, appears as the sister of the *C. tropicalis*–*C. wallacei* pair. *Caenorhabditis tropicalis* was found in our collection in Indonesia and is cosmopolitan in tropical regions. *Caenorhabditis wallacei* was previously found in Bali, Indonesia. The outgroup is *Caenorhabditis doughertyi*, found in South India (Kiontke *et al*. 2011; Félix *et al*. 2014).

### Hybrid crosses that break Haldane's rule

Haldane's rule regarding species hybrids says that individuals of the heterogametic sex are the first affected in hybrid crosses (Haldane 1922), whether by lethality, slow growth, or sterility. Asymmetric results may in addition be obtained between the 2 cross directions [Darwin's “corollary” to Haldane's rule; (Turelli and Moyle 2007)]. In *Caenorhabditis*, males are the heterogametic sex (X0), thus according to Haldane's rule are more likely to be affected than the females. Accordingly, a pattern observed in many *Caenorhabditis* species crosses is that only hybrid F1 females are produced in the progeny, as observed in Baird *et al*. (1992), Woodruff *et al*. (2010), Kiontke *et al*. (2011) , Kozlowska *et al*. (2012), and Bi *et al*. (2019). We observed an asymmetric pattern corresponding to Haldane's rule with an excess of females or slow-growing and small males in crosses between *C*. *ceno* females and *C. tribulationis* males (Fig. 3b), or *C.* sp. 41 females and *C. ubi* males (Fig. 3g).

**Fig. 4.**
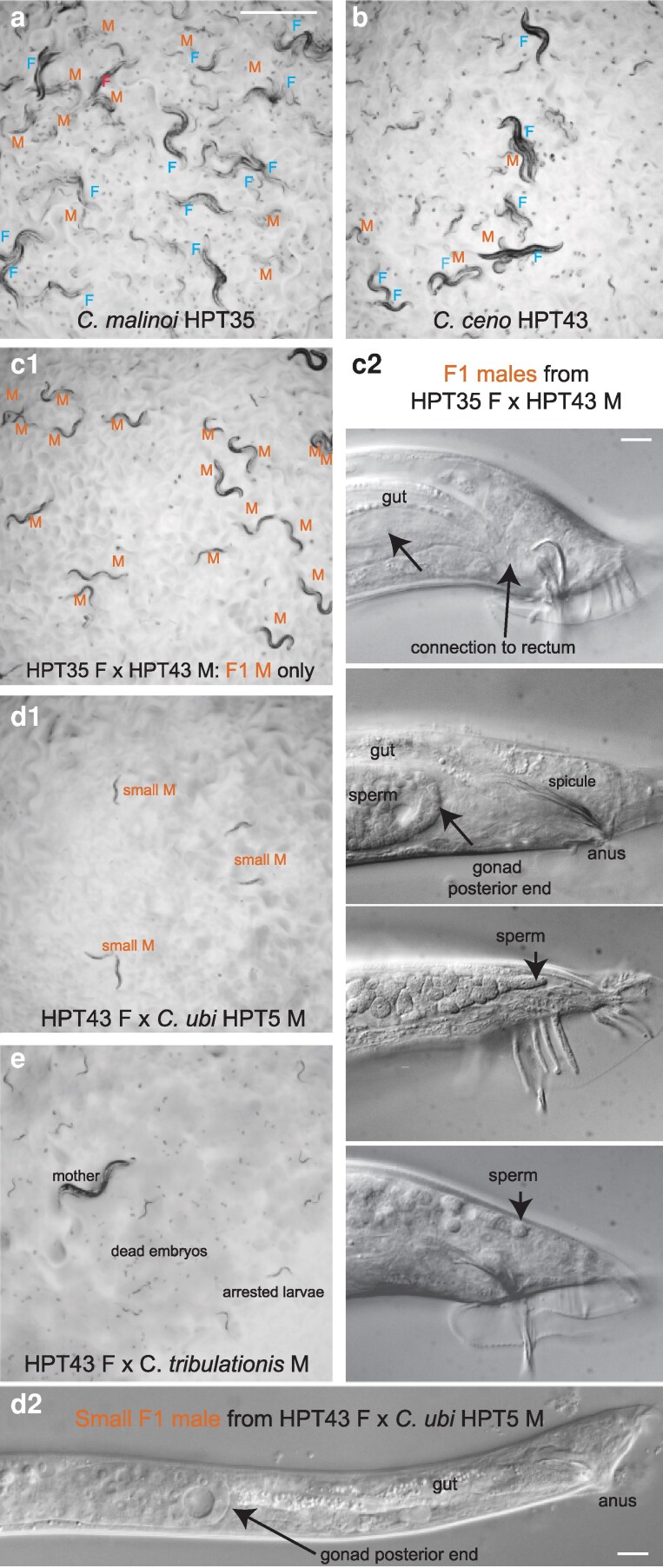
Hybrid crosses between *Caenorhabditis* species break Haldane's rule. Crosses between the *Caenorhabditis* species from South Sulawesi, *C. malinoi* a) and *C. ceno* b), yield males only c), thus breaking Haldane's rule concerning viability. These males are however sterile and display a defective connection between the gonad and the cloaca (rectum), thus preventing the transfer of sperm and of the mating plug material. c2) Nomarski micrographs of F1 hybrid males: on top an apparently normal male and below 3 adult males with a defective gonad–cloaca connection. d) Adult males of small size obtained in the cross of *C. ceno* HPT43 females to *C. ubi* HPT5 males. d1) Shows them on the plate at the same magnification as (a–c). d2) A Nomarski micrograph at the same scale as c2). e) An example of a cross yielding only arrested larvae and many dead embryos, in this case *C. ceno* HPT43 females crossed with *C. tribulationis* JU2774 males. a, b, c1, d1, and e have the same magnification and the corresponding scale bar in a represents 1 mm. Bars in c2 and d2: 10 μm.

However, other crosses instead yielded males only (Supplementary Table 2 and Figs. 3 and 4), for example, the *C. malinoi* females crossed with *C. ceno* males (Fig. 3a), or *C. ceno* females crossed to *C. ubi* males (Fig. 3c). In addition, we less consistently observed a few adult males in some replicates of crosses between *C. brawijaya* females and *C. zanzibari* males (Fig. 3e) and *C. tribulationis* females and *C. ubi* males (Fig. 3f). In some cases, the males were abnormally small (Fig. 4). We focused on the new species pair *C. malinoi* and *C. ceno*, collected a few kilometers apart in South Sulawesi. The result was consistent when multiple strains of *C. malinoi* and *C. ceno* were tested (Supplementary Table 2). In the case of these 2 species, the numerous and normal-size F1 males could not sire progeny and did not deposit a mating plug on females. When observed in the compound microscope, many individuals showed a defective connection between the gonad and the rectum (Fig. 4).

### Partial fertility of hybrids between *C.* sp. 41 and *C. ubi*

Studying genetic incompatibility using genetic mapping is possible when the first-generation (F1) progeny can produce second-generation (F2) progeny, in which reassortment of chromosomes and recombination may have occurred. We found such a case here with *C. ubi* from a forest in East Java and *C.* sp. 41 from the Solomon Islands.

The cross of *C.* sp. 41 BRC20276 females with *C. ubi* HPT5 males—let us call this direction 41ubi—gave rise mostly to F1 females, following Haldane's rule (Fig. 3g, left). These 41ubi F1 females did not give rise to any progeny when placed with males of either parental strain.

**Fig. 5.**
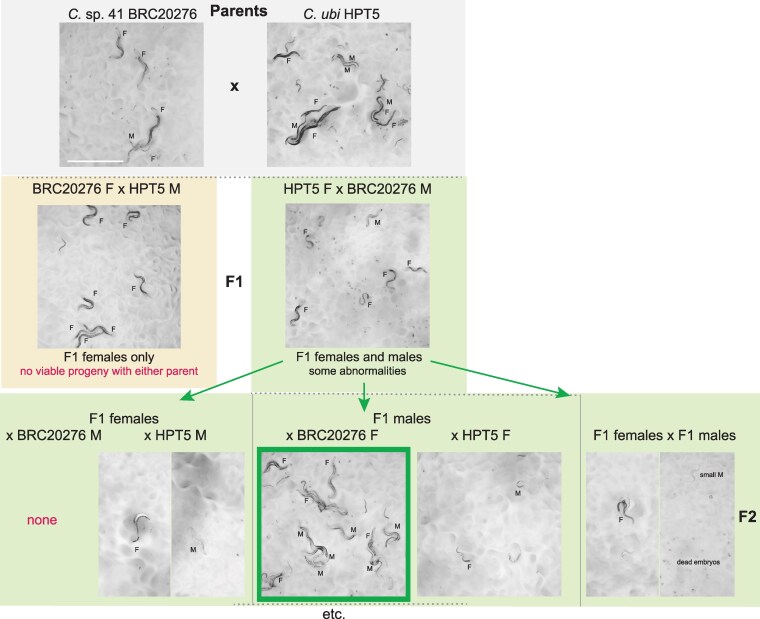
Pictures of the progeny of crosses between *C. ubi* HPT5 and *C.* sp. 41 BRC20276, yielding partially fertile hybrids. Animals of the parental, F1, F2, and backcrosses are shown. In this experiment, only the backcross of the F1 hybrid males with BRC20276 females yielded abundant fertile progeny. The crosses are schematized in Fig. 3g. F, female; M, male. Bar: 1 mm. Same magnification for all panels. Note that the results of the backcrosses with F1 males can yield different results due to within-strain polymorphisms in incompatibility loci in the parental strains.

By contrast, reverse crosses between *C. ubi* HPT5 females and *C.* sp. 41 BRC20276 males (ubi41) yielded partially fertile F1 adults of both sexes, with the males being most fertile (Fig. 3g, right). When crossed to each other, these F1 hybrids yielded few abnormal F2 progeny and rarely could the population propagate. The F1 hybrid females crossed with *C.* sp. 41 BRC20276 males did not give progeny but yielded some progeny when backcrossed to *C. ubi* HPT5 males (Fig. 5). In several replicate experiments (e.g. Fig. 5), when crossed to HPT5 females, the F1 hybrid males gave rise to few small and sterile adult progeny of both sexes and, most interestingly, when crossed to BRC20276 females, could yield fertile progeny. The lineages could be continued for several generations. These crosses also gave rise to many dead embryos and arrested larvae, including what appeared to be a predauer or dauer-like stage.

The parental strains are isofemale lines but not isogenic. In experiments performed on a new thaw of the strains, the backcross of ubi41 F1 males to HPT5 females gave abundant progeny of both sexes, while that to BRC20276 mostly gave rise to males (2 replicates each). The difference with the result shown in Figs. 3 and 5 may be explained by polymorphisms for incompatibility segregating in one or both parental strains (Cutter 2012). In any case, the initial cross using *C. ubi* females and *C.* sp. 41 males followed by a backcross of ubi41 F1 males appears to be a good strategy to produce hybrids between the 2 species.

Thus, *C. ubi* and *C.* sp. 41 are distinct biological species but can yield fertile lineages. This partial reproductive isolation is ideal for studies of incompatibility between closely related species and thus studies of genetic and molecular mechanisms of speciation.

## Discussion

### Biodiversity and biogeography

The present study found 11 different *Caenorhabditis* species in Indonesia, 10 of which could be kept in culture and frozen, forming a collection of 62 strains. Indonesia is rich in species of the *Elegans* group: all collected species were from this group and represent half of the known *Elegans* group species (Fig. 2). Of the 5 previously found species, all occur on several continents, including the 3 selfing species.

Five species of the *Elegans* group are newly described here. *Caenorhabditis indonesiana* is a close relative of *C. wallacei* and *C. tropicalis* and has been found on 3 Indonesian islands. The 4 others were each found on a single island and belong to the *Sinica* subclade of the *Elegans* group that includes *C. sinica*, *C. zanzibari*, *C. tribulationis*, and *C.* sp. 41. This *Sinica* subclade has been found in China (*C. sinica*), Australia (*C. tribulationis*), Solomon Islands (*C.* sp. 41), and islands East of Africa in the Indian Ocean (*C. zanzibari*). Besides geography, this species group is characterized by the trilobed shape of the male sensory organ located anterior to the cloaca called the hook (Kiontke *et al*. 2011; Huang *et al*. 2014; Stevens *et al*. 2019; see Supplementary Fig. 2 legend). Altogether, Indonesia appears to harbor species belonging to several subclades of the *Elegans* group and many potentially endemic species, particularly in 2 subclades. This is consistent with the possibility that this region of the world has been a source of diversification of the *Elegans* group of *Caenorhabditis* species (Kiontke *et al*. 2011; Frézal and Félix 2015).

*Caenorhabditis elegans* seems to fare less well than others at high temperatures in the laboratory (Félix and Duveau 2012; Begasse *et al*. 2015; Frézal *et al*. 2023) and in the field (Kiontke *et al*. 2011; Crombie *et al*. 2019). In this equatorial region, it was found here only once at a high elevation (2,156 m). Consistent with their sampling at higher elevation (1,500–2,400 m), cultures of *C. elegans* HPT48 and *C. brawijaya* HPT49 and HTP50 cannot grow beyond a few generations at 25°C on *E. coli*  OP50 while they thrive at 20°C.

### Violations of Haldane's rule

Our crosses of species within the *Sinica* subclade yielded interesting patterns in the hybrids, the first one being the preferential occurrence of F1 males in some crosses, another providing a pair of species that can serve for genetic studies of incompatibilities arising between closely related species.

Haldane's “rule” corresponds to an overwhelming pattern in species hybrids across organisms, where the heterogametic sex is the first affected in terms of lethality, growth, or sterility. In *Caenorhabditis* species where males carry a single X chromosome, a mechanism explaining the Haldane pattern is an incompatibility between the maternal X chromosome in the hemizygous state (single copy in X0 males) and the rest of the genome in the heterozygous state, while it is rescued by the second X chromosome of the other parent in females (Laurie 1997; Cutter 2024; Li *et al*. 2024). In a recently studied case between *C. briggsae* and *C. nigoni*, *xol-1*, a key gene in sex determination and dosage compensation located on the X chromosome, is insufficiently expressed in sterile hybrid males (Li *et al*. 2024). An alternative or more extreme mechanism for Haldane's rule is sexual transformation of these X0 animals in females (Baird 2002). Additional mechanisms may be at play (Laurie 1997; Cutter 2024).

Any biological “rule” suffers exceptions. Here, we find crosses that contradict the Haldane pattern and only yield hybrid males. Such examples have been found in insects (Laurie 1997); for example, in fruitflies, a cross of *Drosophila simulans* females with *Drosophila melanogaster* males results in hybrid males dying as larvae while females die as embryos (Sawamura *et al*. 1993). In this case, a mismatch between the mother's cytoplasmic content and the mitotic segregation of the father's X chromosome in the early embryo is responsible for the embryonic lethality of females (Ferree and Barbash 2009). Several mechanisms may in principle explain a violation of Haldane's rule: (1) as in this *Drosophila* example, an incompatibility between the paternal X chromosome and the maternal autosomes or cytoplasmic content (e.g. mitochondria, small RNAs, maternal content of proteins), preventing development or fertility of females; (2) incompatibility between the 2 X chromosomes, masculinization of XX animals, and/or a defect in dosage compensation of XX females; (3) in nematodes with X0 males, loss of 1 X chromosome either by selection of sperm not bearing it or by mispairing in oogenesis after fertilization. In addition, we cannot rule out a novel sex determination system, for example, ZZ/ZW (Hodgkin 2002).

In the examples found here in *Caenorhabditis*, the reverse crosses did not yield any adult animals (Fig. 3), which is compatible with several of the above scenarios, including the paternal X-autosome incompatibility if the paternal X acts dominantly in females. In addition to the X chromosome, mitochondria and sex determination loci are both likely to evolve fast in these species and are thus prime candidates for such “non-Haldanian” patterns. We note that the 2 crosses yielding males have a *C. ceno* parent but as males with *C. malinoi* females, and as females with *C. ubi* males. This is consistent with the possibility of a defective dosage compensation in females in the presence of an X chromosome from *C. ceno*, instead of being aberrantly activated in males as in the *C. briggsae–C. nigoni* case (Li *et al*. 2024). Further studies aimed at understanding this exception to Haldane's rule may particularly benefit from the *C. ceno–C. malinoi* pair of sister species, isolated 30 km from each other and yielding many males of normal size.

### Gonad–proctodeum connection as a weak point in male development?

The *C. ceno–C. malinoi* cross reproduces a defect previously seen in *C. remanei*–*C. latens* crosses, where in one cross direction, the F1 males are sterile, and the male sterility is explained by a defective gonad–cloaca connection (Dey *et al*. 2014). We observed such defects in F1 males after crossing *C. ceno* males to *C. ubi* males (Fig. 3) as well as rare F1 males obtained by crossing *C. tribulationis* females to *C. ubi* males (Supplementary Table 2). In the latter case, we observed that the gut connection to the rectum was also defective, resulting in constipated small animals that could not feed normally. *Caenorhabditis remanei* and *C. latens* are not particularly closely related to these *Sinica* subgroup species. We thus propose that gonad connection driven by the migration of the somatic linker cell may be a weak point in *Caenorhabditis* male development. Mutants defective in this connection have been found in *C. elegans* (Palmer *et al*. 2002; Kato and Sternberg 2009; Kato *et al*. 2021; Smith *et al*. 2024). A possibility is that this defective gonad–proctodeum results in multiple species from aberrant dosage compensation in males or a partial sexual transformation affecting this developmental trait more than others.

### A pair of partially cross-fertile species

*Caenorhabditis ubi* and *C.* sp. 41 provide a new case of partially fertile hybrids in the genus, with a pattern distinct from the pairs *C. briggsae*–*C. nigoni* and *C. remanei–C. latens*. Here, the successful cross at the second generation in the experiments shown in Figs. 3 and 5 is a backcross of F1 males, which can further produce progeny. This cross-fertility opens the door to further genetic tests and recombinant mapping. Two other backcrosses yielded none or far fewer progeny (Fig. 5); however, they could also be propagated after a bottleneck.

The most restrictive parental cross (41ubi) gave rise mostly to F1 females, abiding by Haldane's rule. The cross of *C. ubi* females with *C.* sp. 41 males (ubi41) yields both females and males. The ubi41 F1 females could be mated, produce embryos and a few adult progeny with the maternal *C. ubi* parent. By contrast, the ubi41 F1 males appeared more fertile than the females. This can be considered to contradict Haldane's rule in terms of sterility of these ubi41 hybrids; arguably, it may or may not correspond to a physiological sterility of the F1 females since they form F2 embryos, which generally arrest.

This pattern may suggest an X chromosome effect where the *C.* sp. 41 (X41) chromosome in the hemizygous state is incompatible with *C. ubi* autosomes, producing the lethality of the 41ubi F1 males bearing a single X41. Other explanations are possible, such as feminization of hybrid X0 animals bearing the X41 chromosome (Baird 2002; Cutter 2024). An X41 chromosome effect may also explain the greater fertility of the ubi41 F1 males with a single X chromosome from *C. ubi*, compared with their sisters that carry an X chromosome from both parental species. In this case, the X41 would act dominantly on fertility in females. The difference between crosses of *C.* sp. 41 females to *C. ubi* versus to ubi41 F1 males (both with the *C. ubi* X chromosome), where males carrying a hemizygous X41 develop in the latter case only, could be explained by the difference in autosomal content.

What cannot be explained with an X41-autosome incompatibility is that the ubi41 F1 males with Xubi are less cross-fertile with *C. ubi* females than with *C.* sp. 41 females, at least in the series of experiments shown in Figs. 3 (above the dash line) and 5. A simple mitochondrial or maternal effect is not an obvious explanation since their mother is *C. ubi*. A paternal effect of the *C.* sp. 41 fathers could be involved. Multiple incompatibilites and/or sex transformations may coexist, resulting in the observed pattern. In addition, as mentioned above, the parental strains are not isogenic and likely segregate genetic variation for these incompatibilities. Further genetic studies with genotyping will be required to solve this genetic puzzle and address genetic incompatibility mechanisms.

## Supplementary Material

jkaf134_Supplementary_Data

## Data Availability

Strains are available upon request. The ribosomal DNA ITS2 sequences are deposited at GenBank with accession numbers PV569245–PV569249. Sequence reads and transcriptome assemblies are available at NCBI with accession numbers listed in Table 2. The computational pipeline is provided in Supplementary File 1. The phylogenetic tree is provided in Newick format in Supplementary File 2. Supplemental material available at G3 online.
