# Supplementary material for: Five new Caenorhabditis species from Indonesia provide exceptions to Haldane's rule and partial fertility of interspecific hybrids
Source: G3 (Bethesda). 2025 Jun 21;15(8):jkaf134. doi: 10.1093/g3journal/jkaf134 (PMC12341892; doi:10.1093/g3journal/jkaf134)
Supplement: jkaf134_Supplementary_Data [file jkaf134_supplementary_data.zip › Supplemental_Material_Legends_G3-2025-405930.pdf]

Supplemental Information

Figure S1. Location and sample yielding *C. elegans*. For the *C. elegans* isolate, the pictures show the sampling site location at the bottom of the Batok and Bromo volcanoes, the decomposing stems and the laboratory culture (HPT48).

Figure S2. Male tail micrographs of the five newly described species. Nomarski micrographs of individuals of the reference strain. Left: left lateral view. Right: Ventral view. Two different nomenclatures for tail sensory organs are used, one counting nine rays from anterior to posterior, the other distinguishing the ventral from the dorsal ones. In all species, the anterior dorsal ray (ad) is the fifth and the posterior dorsal ray (pd) is the seventh. The precloacal sensillum has a hook shape in all species, and is trilobed at least in all four species of the *Sinica* subclade. We note here that the lateral lobes of this precloacal sensory organ appear to be slightly more dorsal than the central part and could derive from fusion of the cuticle (dorso)-lateral to the central part of the hook (see arrowhead on the *C. ceno* HPT43 ventral view, for which the focal plane was chosen to demonstrate this). We further note that *C. indonesiana* HPT10, outside of the *Sinica* subclade, has a hint of a trilobed hook as well, with the cuticle dorso-lateral to the central part of the hook tending to attach to it. Bar: 10 μm, valid for all panels.

Table S1. Samples and strains. Each sheet corresponds to a sampling location. Each line is a sample, and those positive for *Caenorhabditis* are indicated. Some samples produced two or three different *Caenorhabditis* species. The data are summarized in Table 1.

Table S2. Mating tests. This table shows the result of crossing tests using five L4 stage females and five males. The presence and developmental stage of progeny were assessed over several days. Each tested cell of the matrix is color-coded according to the most advanced developmental stage of the hybrid progeny, as indicated at the bottom of the table. The number indicates the number of independent mating tests, for example '2/2' indicates 2 crosses with the same result; 'n=2-' means two negative crosses without any larval progeny.

Table S3. Links to sequencing data used for the phylogenetic tree.

Table S4. Pairwise distance matrix along branches of the phylogenetic tree.

File S1. Computational workflow for analysis of RNA sequences and phylogenetic reconstruction.

File S2. Phylogenetic tree in Newick format.
